# Stereoselective Anti-Cancer Activities of Ginsenoside Rg3 on Triple Negative Breast Cancer Cell Models

**DOI:** 10.3390/ph12030117

**Published:** 2019-08-01

**Authors:** Maryam Nakhjavani, Helen M. Palethorpe, Yoko Tomita, Eric Smith, Timothy J. Price, Andrea J. Yool, Jinxin V. Pei, Amanda R. Townsend, Jennifer E. Hardingham

**Affiliations:** 1 Molecular Oncology, Basil Hetzel Institute, The Queen Elizabeth Hospital, Woodville South, SA 5011, Australia; 2 Adelaide Medical School, University of Adelaide, Adelaide, SA 5005, Australia; 3 Oncology Unit, The Queen Elizabeth Hospital, Woodville South, SA 5011, Australia

**Keywords:** breast cancer, epimer, stereoselective, Ginsenoside Rg3, triple negative breast cancer

## Abstract

Ginsenoside Rg3 (Rg3) has two epimers, 20(S)-ginsenoside Rg3 (SRg3) and 20(R)-ginsenoside Rg3 (RRg3), and while Rg3 itself has been reported to have anti-cancer properties, few studies have been reported on the anti-cancer effects of the different epimers. The aim was to investigate the stereoselective effects of the Rg3 epimers on triple negative breast cancer (TNBC) cell lines, tested using cell-based assays for proliferation, apoptosis, cell cycle arrest, migration and invasion. Molecular docking showed that Rg3 interacted with the aquaporin 1 (AQP1) water channel (binding score −9.4 kJ mol^−1^). The *Xenopus laevis* oocyte expression system was used to study the effect of Rg3 epimers on the AQP1 water permeability. The AQP1 expression in TNBC cell lines was compared with quantitative-polymerase chain reaction (PCR). The results showed that only SRg3 inhibited the AQP1 water flux and inhibited the proliferation of MDA-MB-231 (100 μM), due to cell cycle arrest at G0/G1. SRg3 inhibited the chemoattractant-induced migration of MDA-MB-231. The AQP1 expression in MDA-MB-231 was higher than in HCC1143 or DU4475 cell lines. These results suggest a role for AQP1 in the proliferation and chemoattractant-induced migration of this cell line. Compared to SRg3, RRg3 had more potency and efficacy, inhibiting the migration and invasion of MDA-MB-231. Rg3 has stereoselective anti-cancer effects in the AQP1 high-expressing cell line MDA-MB-231.

## 1. Introduction

Ginsenosides are a class of natural triterpenoid saponins with the general structure of an aglycone steroid backbone and a glycoside side chain. They are extracted from the plant *Panax ginseng* Meyer, commonly known as ginseng, and play an important role in the medicinal effects of ginseng extract [1,2]. Ginsenoside Rg3 (Rg3) is one of the extensively studied members of the ginsenoside family, having a variety of biological actions and efficacies, including anti-oxidant properties [3] and protective effects in cardiovascular diseases [4,5,6], neurological disorders [7,8,9,10], diabetes [11,12,13], immune function and inflammation [14,15,16,17], and cancer [18]. Although many papers refer to Rg3 as a single molecule and report the effects of Rg3, rather than a specific epimer, it is noteworthy that Rg3, like other ginsenosides, has two epimers: 20(S)-ginsenoside Rg3 (SRg3) and 20(R)-ginsenoside Rg3 (RRg3). Each of these epimers has distinct pharmacological actions, intracellular targets, effects and efficacies. For example, the SRg3 epimer activates caspases in the human gastric cancer cell line [19] and inhibits Ca^2+^, Na^+^ and K^+^ ion channels [20], while the RRg3 epimer has antioxidant properties to combat cyclophosphamide-induced cellular stress [3].

The anti-cancer properties of Rg3 have made it a notable drug candidate for many cancer models. Few studies have focused on the anti-cancer effects of Rg3 in breast cancer models, specifically in triple negative breast cancer (TNBC), a subtype of breast cancer with a poor prognosis [21]. Since this subtype of breast tumour lacks the expression or overexpression of an estrogen receptor, progesterone receptor or human epidermal growth factor receptor (HER) 2, there are as yet no targeted therapies for TNBC; chemotherapy regimens, along with their adverse effects, remain the mainstay of treatment in most TNBC patients. Thus, finding a targeted biological agent for TNBC would make a paradigm shift in the treatment of these patients. Previous reported studies have shown that Rg3-induced apoptosis [22] inhibited the activation of NF-κB [23], induced G0/G1 arrest [24] and inhibited chemoinvasion directed by CXCR4 [25] in breast cancer cell lines. However, these studies did not use a specific epimer of Rg3, nor did they specify the ratio of the two epimers. 

**Figure 1:**
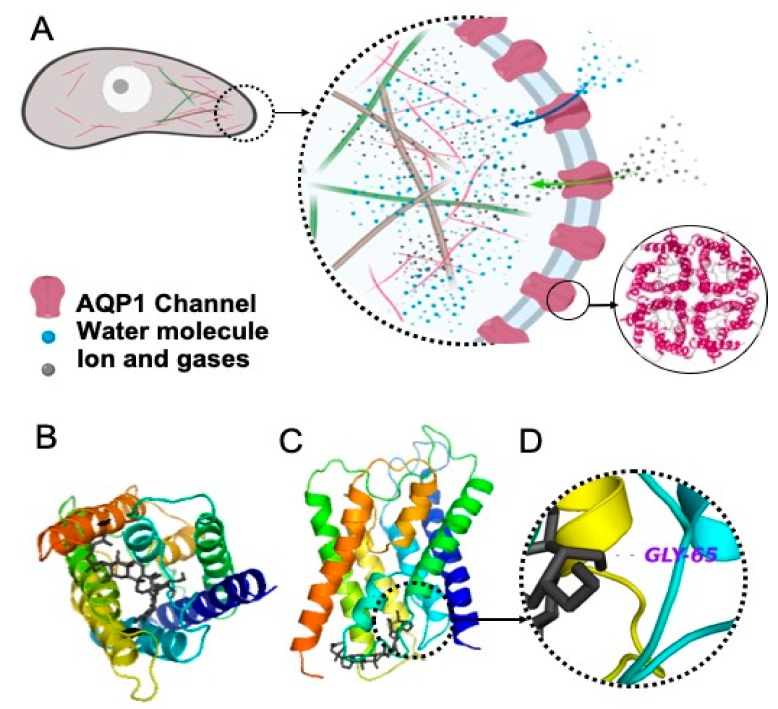
(**A**) The role of aquaporin 1 (AQP1) in cell migration and invasion (as reviewed in [39]). AQPs are redistributed to the leading edge of the migrating cell, leading water, ions and gases inside the cell; hence, along with changes in actin polymerisation, they play a role in the forward movement of the cell. AQP1 is a tetramer. Water passes through the pore of each monomer, and ions and gases pass through the central pore of the tetramer. (**B**) Top view of an AQP1 monomer, being blocked with Rg3, the black structure, (**C**) Side view of an AQP1 monomer, blocked with Rg3, and (**D**) H-bonding between Rg3 and Gly 65.

Aquaporin 1 (AQP1) is a member of the AQP family of water transporters. Like other AQPs, AQP1 is a homo-tetramer. Each monomer, as depicted in Figure 1A, works as a single channel for water transport. The central pore of the tetramer is responsible for gas and ion transport, the latter of which is gated by cGMP. It is already shown that AQP1 plays a role in the growth, angiogenesis and metastasis of tumours [26,27,28,29]. AQP1 is highly expressed in mouse models of breast tumour [30], and AQP1 deficiency in such models decreased the number of lung metastases [31]. Furthermore, clinical studies have shown that some TNBC tumours have higher levels of AQP1 expression and an expression correlated with a poorer prognosis [32,33].

To date, no studies have focused on the stereoselectivity of Rg3 epimers on TNBC cell lines, and no studies have shown the interaction between Rg3 epimers and AQP1. The aim of our study was to investigate the stereoselective effects of Rg3 on human TNBC cell lines. In particular, our aim was to investigate whether these two epimers have effects on different functions on TNBC in cell line models, including proliferation, apoptosis, cell cycle, migration and invasion. Furthermore, in line with our previous research focus [28,34,35,36,37], we investigated the interaction of Rg3 epimers with AQP1 in in silico models.

## 2. Results

### 2.1. Interaction of Rg3 with AQP1

#### 2.1.1. Molecular Docking of Rg3 

The in silico molecular docking studies were performed on Rg3 docked within the water channel of AQP1, AQP2, AQP4 and AQP5. The results in Table 1 are the scores based on Gibbs free energy (kJ mol^−1^). 

The modelled binding energetically favoured AQP1 (−9.4 kJ mol^−1^) at a level comparable to known AQP1-inhibitors such as bacopaside I (−9.2 kJ mol^−1^) and bacopaside II (−9.3 kJ mol^−1^) [38]. Figure 1A illustrates the role of the AQP channels in migration and invasion (as reviewed in [39]). We showed that the water channel of the AQP1 monomer was blocked by Rg3 (Figure 1B,C). The H-bonding between the OH group (located on the C4’ of the second sugar molecule) with Gly^65^ (located in the second transmembrane helices, between loops A and B), with a distance of 3.4 Å, is shown in Figure 1D. 

#### 2.1.2. Stereoselectivity of Rg3 in Inhibiting AQP1 Water Channel

**Figure 2:**
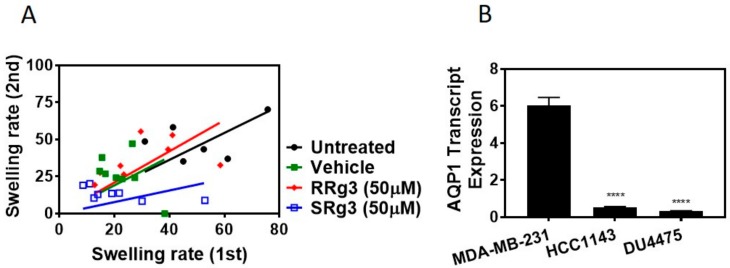
(**A**) Double swelling assay showing the swelling rates for the first and the second swelling on a single oocyte. Eight oocytes per treatment were measured for swelling in a hypotonic medium, before and 2 h after exposure to a vehicle or epimers of Rg3. The results were analysed and presented with a linear regression. (**B**) The AQP1 transcript expression in MDA-MB-231, HCC1143 and DU4475 cell lines. Each data point represents a mean ± SD value of 3 replicates, and comparisons were made with the vehicle control group (**** *p* < 0.0001).

To find out if this interaction of Rg3-AQP1 is stereoselective, a *Xenopus laevis* oocyte expression system expressing human AQP1 was used. Native *Xenopus laevis* oocytes lack water channels, and hence a heterologous expression of human AQP1 on these cells makes them permeable to water. Following exposure to hypotonic media, water penetrates the cells based on osmotic driving forces. Figure 2A shows the result of the double swelling assays. The slope (± standard error) of the swelling rate for untreated, vehicle, RRg3 and SRg3 groups was 0.9 ± 0.1, 0.9 ± 0.2, 1.0 ± 0.2 and 0.4 ± 0.1, respectively. This shows that the rate of swelling in untreated, vehicle or RRg3 treated oocytes was similar, while the rate of swelling for SRg3 treated cells was reduced by almost 2.6 times, indicating the blockage of AQP1 with SRg3. 

### 2.2. Rg3 Has Stereoselectivity and Cell Line-Specificity in Inhibition of Proliferation

**Figure 3:**
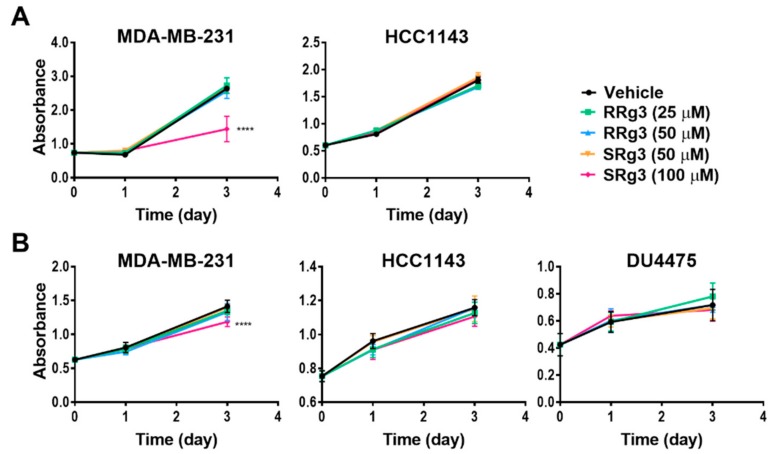
Effect of SRg3 and RRg3 on the proliferation of MDA-MB-231, HCC1143 and DU4475 triple negative breast cancer cell lines, after 3 days, with (**A**) a crystal violet assay on the adherent cell lines and (**B**) an MTS assay. Only 100 µM of SRg3 showed an inhibition of proliferation of MDA-MB-231 in both assays, indicating the cell line selectivity and stereoselective effect of Rg3 for the inhibition of proliferation. Each data point represents a mean ± SD value of 6 replicates, and comparisons are made with the vehicle control group (**** *p* < 0.0001).

To study the effect of Rg3 epimers on the proliferation of TNBC cell lines, MDA-MB-231, HCC1143 and the non-adherent DU4475 were tested. Within 3 days of treatment, all of the cell lines showed an increased cell proliferation. Interestingly, only SRg3 at 100 µM had an anti-proliferative effect on MDA-MB-231 in both assays (Figure 3A,B). A crystal violet assay showed that SRg3 (100 µM) inhibited the proliferation of cells by 45%. This indicates a stereoselective activity and cell line specificity of Rg3, since neither of the other cell lines showed an inhibition of proliferation with SRg3 or RRg3. A potential mechanism for the effect of SRg3 on the inhibition of proliferation in MDA-MB-231 was investigated by measuring the AQP1 transcript expression. Since the MDA-MB-231 cells had an 11 and 19 times higher expression of AQP1 compared to the HCC1143 and DU4475 cell lines, respectively (*p* < 0.0001) (Figure 2B), SRg3 may, in part, exert its activity through blocking AQP1.

### 2.3. Cytostatic Effect of SRg3 Inhibits Cell Proliferation in MDA-MB-231 Cell Line without Inducing Apoptosis

**Figure 4:**
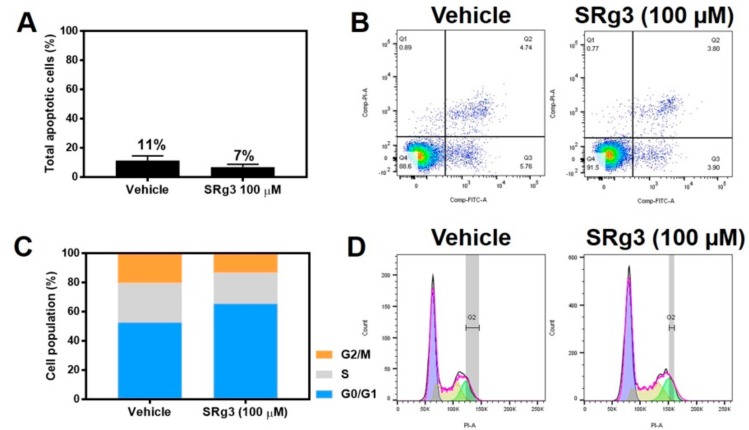
The effect of SRg3 on apoptosis and cell cycle arrest in the MDA-MB-231 cell line. (**A**) Total apoptotic cells (%) induced by vehicle or 100 µM SRg3 after 3 days of exposure; (**B**) Scatter plots of untreated cells or the cells treated with vehicle or SRg3. The left lower quadrant, right lower quadrant, right upper quadrant and left upper quadrant indicate viable cells, early apoptotic cells, late apoptotic cells and necrotic cells, respectively. (**C**) Cell population (%) in each of the G0/G1, S and G2/M phases of the cell cycle. (**D**) Histograms of the untreated, vehicle and SRG3-treated cells, following staining with PI. The violet, yellow and green curves represent events in the G0/G1, S and G2/M phases, respectively. The data presented is representative of 3 repeats.

The MDA-MB-231 cell line was exposed to a concentration of 100 µM SRg3 for 3 days, after which the cells were tested to see if the inhibition of proliferation was due to the induction of apoptosis. As shown in Figure 4A,B, there were no significant differences between the amount of apoptosis induced by vehicle (11.29% ± 3.22) or 100 µM of SRg3 (6.96% ± 1. 81) (*p* = 0.11). Since SRg3 was not inducing apoptosis, the cells were tested to determine if the inhibition of proliferation was due to cell cycle arrest. The percentages of cells in each cell cycle phase for the untreated cells were 51.3% ± 2.9 for G0/G1, 29.8% ± 1.8 for S, and 17.1% ± 1.9 for G2/M (Figure 4C,D). Vehicle-treated cells have similar values, at 52.8 ± 5.1, 27.6 ± 3.0 and 19.8 ± 1.2, respectively. The statistical analysis showed no significant differences between the untreated and vehicle control groups for each phase. However, the cells treated with 100 µM of SRg3 for 3 days showed a significant accumulation of cells in G0/G1 (65.3% ± 3.22) (*p* < 0.0001) and a reduction of cells in the G2/M phase (12.5% ± 1.4) (*p* < 0.05), compared to the vehicle group. Together, these data suggest that 100 µM SRg3 inhibited the MDA-MB-231 proliferation by inducing a G0/G1 cell cycle arrest.

### 2.4. Stereoselective Inhibition of Migration of MDA-MB-231 Cell Line

**Figure 5:**
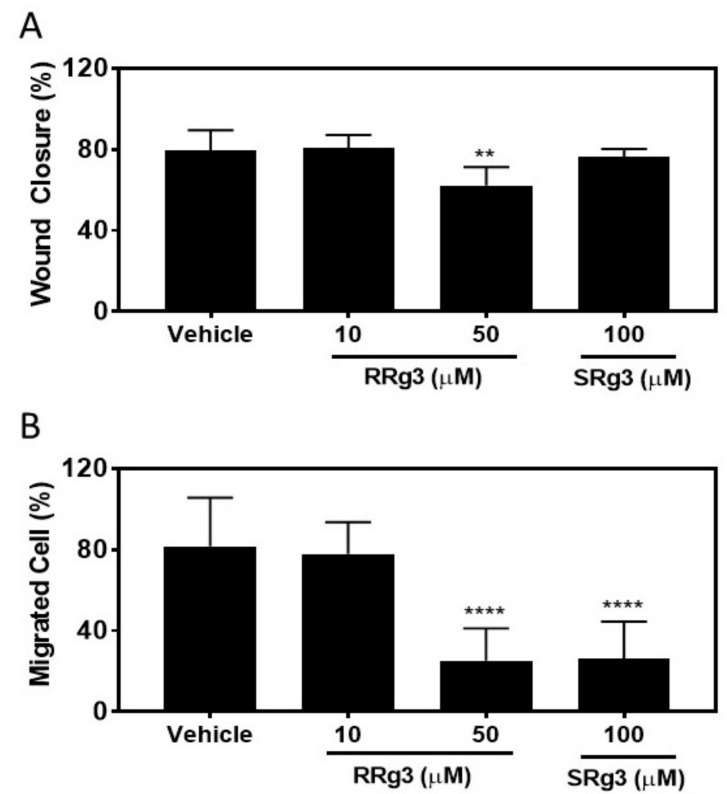
Migration assays on the MDA-MB-231 cell line following exposure to RRg3 and SRg3. (**A**) The percentage of wound closure following exposure of the MDA-MB-231 cell line to RRg3 and SRg3. Data is presented as mean ± SD of 6 repeats, and comparisons are made with the vehicle control group (** *p* = 0.001). (**B**) The transwell migration assay on the MDA-MB-231 cell line following exposure to RRg3 and SRg3. Data are presented as mean ± SD of 3 replicates, and comparisons are made with the vehicle control group (**** *p* < 0.0001).

To study if the epimers of Rg3 had any effect on the MDA-MB-231 migration, the cells were pre-treated with Rg3 epimers for 3 days, after which they were seeded for a circular wound closure assay. The results of this assay showed that after 24 h, the untreated and vehicle cells closed the circular wound by about 80% (Figure 5A). The wound closure in the cells treated with 100 µM of SRg3 was 76% ± 3.8, not significantly different to the vehicle (*p* = 0.81), while the wound closure in the cells treated with 50 µM of RRg3 was inhibited by 22 % compared to the vehicle control group (*p* = 0.001).

We showed, using a chemoattractant transwell migration assay, that at 50 and 100 µM of RRg3 and SRg3, the transwell migration of MDA-MB-231 cells was significantly inhibited by 69 and 68%, respectively (*p* < 0.0001).

### 2.5. Stereoselective Inhibition of Invasion

**Figure 6:**
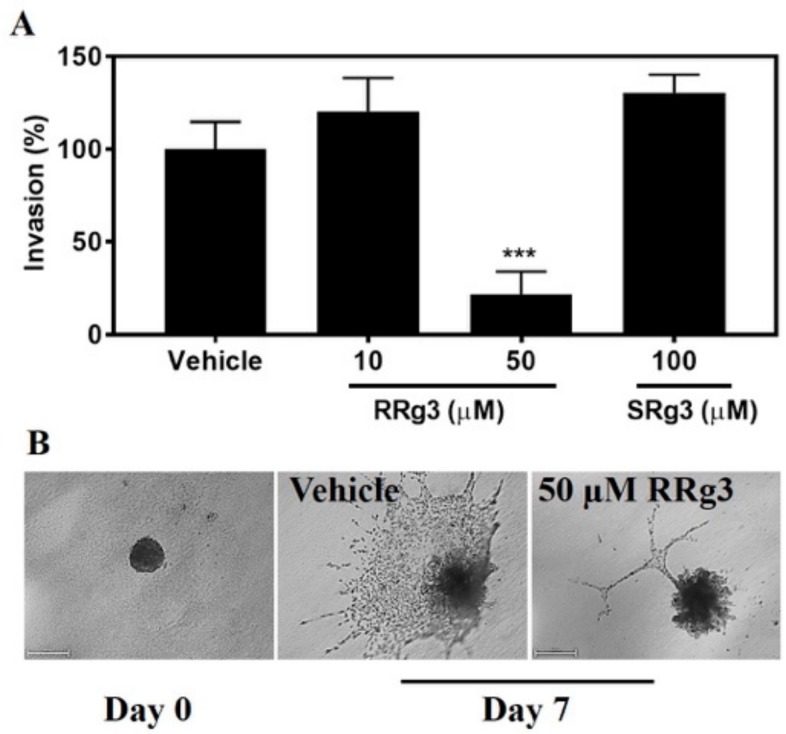
Spheroid invasion assay on the MDA-MB-231 cell line following exposure to RRg3 and SRg3. (**A**) The percentage of increase in the area, as an indicator of invasion to the extracellular matrix, following exposure of the MDA-MB-231 spheroids to RRg3 and SRg3. Data are presented as mean ± SD of 3 replicates, and comparisons are made with the vehicle control group (***) *p* = 0.0001. (**B**) Representative images of the vehicle control and RRg3-treated spheroids after 7 days, indicating the inhibition of spheroid invasion in the RRg3-treated spheroids.

The ability of epimers of Rg3 to inhibit invasion was tested with a spheroid invasion assay. As shown in Figure 6, 50 µM of RRg3 resulted in inhibition (*p* = 0.0001). The inhibition of invasion was 78% for the spheroids treated with 50 µM RRg3. SRg3 did not inhibit spheroid invasion in this assay. 

## 3. Discussion

This is the first study to investigate the stereoselective effects of epimers of Rg3 on TNBC cell lines. To our knowledge, the literature has no comparable studies that define the Rg3 epimers, or the specific ratio of SRg3/RRg3 in breast cancer models. Notably, we showed the stereoselectivity of Rg3 in the inhibition of proliferation in MDA-MB-231, the only cell line that showed sensitivity toward the anti-proliferative effects of SRg3. Neither of the Rg3 epimers inhibited the proliferation of the HCC1143 or DU4475 cell lines. MDA-MB-231 is a basal-like B [40,41] but with claudin-low [42] or mesenchymal-like [41] features, representative of tumours with a worse prognosis and more aggressive nature. Higher levels of AQP1 expression in breast tumours have been correlated with a triple negativity, poorer prognosis of the disease and higher tumour grade [33,43]. We showed that the AQP1 expression in MDA-MB-231 is much higher than in the HCC1143 and DU4475 cell lines. Molecular docking studies showed that Rg3 had a promising binding score with AQP1, comparable with some other blockers of AQP1 such as AqB013 [44], bacopaside I and bacopaside II [38]. Furthermore, for the first time, we showed that SRg3 was the only epimer that inhibited the AQP1 water flux. This stereoselective inhibition of the AQP1 water flux and inhibition of proliferation in the cell line with a higher expression of AQP1 by SRg3 suggests that AQP1 might be one of the important proteins involved in the proliferation of MDA-MB-231. Importantly, it is already reported that the over-expression of AQP1 in MDA-MB-231 significantly increased the proliferation and chemotactic invasion [45].

We also showed that the inhibition of proliferation of MDA-MB-231 by SRg3 was not due to the induction of apoptosis, but rather due to cell cycle arrest at the G0/G1 phase of the cell cycle. This G0/G1-arrest mechanism was comparable with similar studies in prostate [45], melanoma [46] and breast cell lines [24]. None of them, however, have defined a specific epimer in their studies. For example, the study on the breast cancer cell line exposed MCF7 (an estrogen and progesterone receptor positive cell line) to Rg3 and the heated extract of ginseng, containing about 5% Rg3 [24]. We have shown that it is SRg3 that causes G0/G1-arrest in MDA-MB-231. In fact, it has been shown that the over-expression of AQP1 causes a higher level of cyclin D and E, which are crucial for phase transition [47]. Cyclin E is a regulator for G1-S transition, and the blockage of AQP1 or inhibiting the expression of AQP1, as suggested by Pan et al. [45], inhibits the G1-S transition and arrests the cells in G0/G1.

Migration was tested in two assays. While the scratch wound closure assay measures the rate of cells migrating on plastic to close the circular wound, the transwell migration assay measures the ability of cells in suspension to migrate toward a chemoattractant [48]. Both of the epimers inhibited the chemoattractant-induced migration of cells. Notably, SRg3, which inhibited the water transport function of AQP1 in a stereoselective manner, was only effective in the chemoattractant-induced migration of cells as opposed to adherent cells migrating on plastic. This suggests that these epimers might have different mechanisms of action for the inhibition of migration. For example, in ovarian carcinoma cell lines, SRg3 was the only epimer that inhibited migration and invasion via blocking hypoxia-induced epithelial-mesenchymal transition (EMT), the degradation of hypoxia-inducible factor-1α (HIF-1α) and the transcriptional repression of Snail and hence E-cadherin [49]. Importantly, Rg3 decreased the expression of AQP1 in the PC-M3 prostate cancer cell line, causing the inhibition of the chemoattractant-induced migration of these tumour cells [45]. It is already known that AQP1 is involved in the chemotactic migration of the cancer cells [29,50,51]. The chemotactic movement of the cancer cells is an important driver toward metastasis. Cancer cells sense the chemotactic gradient, and polarize into the leading edge to move forward toward the chemotactic agent. AQP1 is found on the leading edge of migrating cells (Figure 1A). AQP1, via directing the water influx and interactions with the actin cytoskeleton at the protrusion site of the migrating cell, plays roles in the migration of cancer cells, as reviewed in [39]. In our studied assays of migration and invasion, the transwell migration assay was the only assay in which the chemoattractant-induced migration of cells was assessed and SRg3, as a stereoselective inhibitor of AQP1, showed inhibitory effects. This suggests that AQP1, along with other mechanisms, is involved in the chemoattractant-induced migration of highly AQP1-expressing MDA-MB-231 cells. 

While SRg3 showed no efficacy in the inhibition of migration in the wound closure migration assay nor in the spheroid invasion assays, RRg3, with a higher potency, inhibited migration and invasion in all of the studied assays. This suggests that RRg3 modulates different targets and pathways. Similar to our results, studies on the lung cancer A549 cell line showed that RRg3 at concentrations < 50 µg/mL did not inhibit the proliferation of the cells, but, in a stereoselective manner, suppressed TGF-β1-induced EMT, through repressing the Snail expression and inhibiting the activation of Smad and non-Smad (p38 MAPK) signalling pathways, hence inhibiting the E-cadherin expression [52]. The RRg3 inhibition of TGF-β1-induced EMT caused the inhibition of migration, invasion and anoikis resistance. RRg3 inhibited the TGF-β1-induced MMP-2 expression and inhibited the activation of Smad2 and p38 MAPK [52]. Other suggested mechanisms for the RRg3 inhibition of EMT and invasion were through the downregulation of fucosyltransferase IV (FUT4) [53]. FUT4 is an enzyme responsible for abnormal fucosylation in cancer cells, associated with the proliferation and metastasis of breast tumour cells [54], and it is also suggested as a biomarker for the diagnosis of breast tumours [55]. 

This is the first paper to study the stereoselectivity of epimers of Rg3 in TNBC cell lines and demonstrates that SRg3 and RRg3 have distinct effects. Furthermore, this is the first time that the interaction between Rg3 epimers and AQP1 is demonstrated. The effect of Rg3 epimers on the inhibition of proliferation of TNBC cell lines was specific to MDA-MB-231, suggesting that basal-like claudin-low or mesenchymal type tumours, with a high AQP1 expression, might be better candidates for treatment with SRg3. SRg3 had cytostatic effects on the MDA-MB-231 cell line, leading to the inhibition of proliferation. It inhibited the chemoattractant-induced cell migration and, notably, was the only epimer that blocked the AQP1 water channel function. This suggests that the SRg3 blocking of the AQP1-mediated water flux is a potential contributor to the mechanism of inhibition of chemoattractant-induced cell migration and the inhibition of proliferation in this cell line, but may not be the only target of action. Importantly, RRg3 is not cytotoxic to the cells, yet it inhibited the cell migration and invasion of MDA-MB-231, with a higher potency. The distinct and stereoselective actions of each epimer suggest that SRg3 and RRg3 should be considered as separate drug candidates. Although this study was limited to cell lines and in vitro assays, these results will inform the doses of each epimer to be tested in a mouse model of breast cancer.

## 4. Materials and Methods 

### 4.1. Materials

SRg3 (Sigma-Aldrich, St Louis, MO, USA) and RRg3 (AdooQ Bioseciences Irvine, CA, USA) epimers, both with purities > 98%, were dissolved in dimethyl sulfoxide (DMSO) at 12.7 and 6.5 mM stocks and stored in aliquots at −20 °C. Due to the low water solubility, log S −4.04 (ChemAxon, Cambridge, MA, USA), and relatively high lipophilicity (logP 4) of Rg3, these stocks were found to have the highest stock concentration of Rg3, which did not precipitate out upon dilution in aqueous media. The maximum concentration of DMSO with no observable biological effects in this study was found to be 0.8%. Triple negative breast cancer cell lines; MDA-MB-231 (basal-like with mesenchymal or claudin-low phenotype), HCC1143 (basal-like), and DU4475 (basal-like) were purchased from the American Type Culture Collection (ATCC; Manassas, VA, USA) and used at low passage numbers. 

### 4.2. Molecular Docking of Rg3

The molecular docking of Rg3 on aquaporin (AQP) channels was performed as previously described [38,56]. The crystal structures of the proteins were obtained from the protein data bank of NCBI (RCSB PDB). The structure IDs were as follows: AQP1 (1FQY), AQP2 (4NEF), AQP4 (3GD8), AQP5 (3D9S). The SMILES structure of Rg3 was obtained from PubChem. The three-dimensional structure of Rg3 was prepared in the UCSF Chimera program (version 1.13.1-mac64). The Autodock Vina algorithm (version 1.1.2_Mac) and UCSF Chimera program were used for in silico molecular docking. Images were prepared in the PyMol Molecular Graphics System, The X Window System, XQuartz 2.7.11. Notably, Autodock uses a stochastic search method to explore the conformational space of the ligand molecule, and this is by the random generation of distinct conformations, leading to finding a global energy minimum, expressed by a score for the Gibbs free energy of protein-ligand binding [57]. Due to this random generation of conformations, it is not practical to study a single epimer with this algorithm.

### 4.3. Oocyte Expression System and Swelling Assay

The unfertilized oocytes from a native *Xenopus laevis* frog were prepared and maintained, as previously described [38]. Briefly, the oocytes were injected with 3 ng of AQP1 cRNA and incubated at 16–18 °C for 3 days to allow for AQP1 expression. The inhibitory effect of Rg3 epimers on the AQP1 water channel was measured with the double-swelling assay. The swelling rate in hypotonic media (50% saline) for each oocyte was recorded and measured with ImageJ software (Wayne Rashband, National Institutes of Health, Bethesda, MD, USA), before and after a 2 h exposure to normal saline (as the untreated group), vehicle, or 50 µM of RRg3 or SRg3. Each treatment group consisted of 8 oocytes, and the rate of swelling in each group was compared with the vehicle group, using a linear regression analysis, as previously described [38].

### 4.4. Cell Culture

All of the TNBC cell lines were cultured as recommended by ATCC. The cells were thawed, and cultured in their respective media supplemented with a final concentration of 10% foetal bovine serum (FBS; Corning, Corning, NY, USA), 1% penicillin-streptomycin solution (Life Technologies, Grand Island, NY, USA) and 1% GlutaMax (Life Technologies), and they were incubated at 37 °C, 5% CO_2_ in the air. 

### 4.5. Quantitative PCR for Expression of AQP1

The cell lines were seeded at 5 × 10^5^ cells/well in 6-well plates. Following an overnight incubation, RNA was extracted using the PureLink RNA mini kit (Life Technologies), followed by the reverse transcription of 200 ng RNA with the iScript cDNA Synthesis Kit (Bio-Rad Laboratories, Hercules, CA, USA). The duplex TaqMan Gene Expression Assays for aquaporin-1 (AQP1; Hs01028916_m1; Applied Biosystems, Foster City, CA, USA) and the reference gene serine-rich coiled-coil domain-containing protein 2 (CCSER2; HS00982799_mH, Applied Biosystems, Foster City, CA, USA) were used to determine the transcript expression, as previously described [58]. Reactions were performed using the Applied Biosystems ViiA 7 Real-Time PCR System (Life Technologies) with activation for 30 s at 95 °C, followed by 40 cycles of 15 s at 95 °C and 30 s at 60 °C. The AQP1 transcript expression was calculated using the 2^-ΔCt^ formula.

### 4.6. Proliferation Assay 

The effect of Rg3 epimers on the proliferation of the MDA-MB-231 and HCC1143 adherent cell lines was tested with a crystal violet assay, as described previously [58]. DU4475, a non-adherent cell line, along with the two adherent ones, were also tested with the MTS assay (CellTiter 96^®^ AQueous Non-Radioactive Cell Proliferation Assay, Promega, Madison, WI, USA), as described previously [34]. Briefly, 7 × 10^3^ cells/well were seeded in 96-well plates, incubated overnight and treated with 0–100 µM (final concentration) Rg3 epimers (6 replicates). The absorptions at 595 nm (for the crystal violet assay) and 490 nm (for the MTS assay) were measured at 0, 24 and 72 h of treatment. 

### 4.7. Apoptosis Assay

An apoptosis assay was performed using the Annexin-V-FLUOS staining kit (Roche Diagnostics, Mannheim, Germany), based on the previously described method [34]. Briefly, a density of 1 × 10^5^ cells/well of 6-well plates were seeded in triplicate and incubated overnight. MDA-MB-231 cells were treated with 100 µM SRg3 for 72 h. Paclitaxel (400 nM) was used as a positive control. A control for necrosis was prepared by heating the cells at 63 °C for 30 min. Following the staining of the samples, they were analysed in BD FACSCanto II (BD Biosciences, San Jose, CA, USA) and FlowJo software, v 10.4 (FlowJo, LLC, Ashland, OR, USA).

### 4.8. Cell Cycle Analysis

MDA-MB-231 cells were seeded at 1 × 10^5^ cells/well of 6-well plates, in triplicate. After an overnight incubation, 100 µM of SRg3 was added to the cells. After 3 days, propidium iodide staining and a cell cycle analysis were performed on the cells, as previously described [59,60]. The samples were analysed with BD FACSCanto II and FlowJo software, v 10.4.

### 4.9. Scratch Wound Closure Assay

MDA-MB-231 cells were pre-treated with Rg3 epimers for 3 days and then seeded at 8 × 10^4^ cells/well in 96-well plates for a scratch wound closure assay. The cells were exposed to different concentrations of RRg3 and SRg3, and the assay was performed as described previously [61]. Images were taken at time 0 and 24 h using a Nikon microscope, and the wound closure (%) was measured using ImageJ software. The relative wound closure (%) at time 24 h was calculated compared to time 0, with 6 replicates. 

### 4.10. Transwell Migration Assay

MDA-MB-231 cells were pre-treated with either vehicle or Rg3 epimers for 3 days. Then, 1 × 10^5^ cells were suspended in 250 µL of serum-free DMEM containing vehicle or Rg3 epimers and placed in the upper chamber of the Corning^®^ transwells (8 µm pore size). The lower chamber was filled with 750 µL DMEM supplemented with final concentrations of 10% FBS, 1% penicillin-streptomycin solution and 1% GlutaMax. The cells were incubated for 4.5 h, after which the cells on top of the membrane were removed with a cotton swab. The migrated cells on the other side of the membrane were fixed in 10% neutral buffered formalin for 30 min, stained in a crystal violet solution (1% crystal violet in 2% ethanol) for 10 min, and washed in distilled water. The experiment was carried out in triplicate, and the total migrated cells were counted in five fields of view per chamber, at 200× magnification using NIS-Elements (Nikon, Tokyo, Japan). The migration percentage for each treatment group is presented relative to the average vehicle control group.

### 4.11. Spheroid Invasion Assay

A single cell suspension (3 × 10^3^ cell/well) and 1X Spheroid Formation ECM (Cultrex^®^, Trevigen Inc., Gaithersburg, MD, USA) (5 µL/well) was prepared, and 50 µL of this suspension was placed in each well of the 96-well ultra-low attachment Costar^®^ plates (Corning Inc., Corning, NY, USA). The plate was centrifuged at 200× *g* for 3 min and incubated in a 37 °C, 5% CO_2_ incubator for 72 h. Then, the plate was left on ice for 15 min, and 50 µL of Invasion Matrix (Cultrex^®^) was added to each well (day 0). The plate was then centrifuged at 300× *g*, 4 °C, 5 min, followed by a 1 h incubation. The spheres were then treated with different concentrations of Rg3. At day 0 and day 7, images were taken of the spheres using a Nikon Eclipse TE2000-U light microscope, and the area of each sphere was measured using NIS-elements software (Nikon, Tokyo, Japan). The invasion (%) of each spheroid was normalized to the mean invasion area of the vehicle group.

### 4.12. Statistical Analysis

A one-way or two-way analysis of variance (ANOVA) was performed for the data analysis using GraphPad Prism (version 7.02). The data are presented as the mean ± standard deviation (SD). *p* < 0.05 was considered as the level of statistical significance.

## Figures and Tables

**Table 1:** The results of the in silico molecular docking of Rg3 with aquaporin water channels in comparison with other blockers of AQP1. The results are presented as Gibbs free energy (kJ mol^−1^).

Molecule	Binding Score (kJ mol^−1^)			
	AQP1	AQP2	AQP4	AQP5
Ginsenoside Rg3	−9.4	−6.4	−6.1	−4
Bacopaside I	−9.2 [38]	7.4	−5.2	−6.9
Bacopaside II	−9.3 [38]	2.2	−5.2	−6.4
